# Arthropod diversity in the alpine tundra using metabarcoding: Spatial and temporal differences in alpha‐ and beta‐diversity

**DOI:** 10.1002/ece3.10969

**Published:** 2024-02-09

**Authors:** Nils Hein, Jonas J. Astrin, Niklas Beckers, Hendrik Giebner, Kathrin Langen, Jörg Löffler, Bernhard Misof, Vera G. Fonseca

**Affiliations:** ^1^ Leibniz Institute for the Analysis of Biodiversity Change (LIB) Bonn Germany; ^2^ Department of Geography University of Bonn Bonn Germany; ^3^ Centre for Environment Fisheries and Aquaculture Science (Cefas) Weymouth UK

**Keywords:** Arctic–alpine ecosystems, arthropods, biodiversity, ethanol‐based DNA, microclimate, Scandinavia

## Abstract

All ecosystems face ecological challenges in this century. Therefore, it is becoming increasingly important to understand the ecology and degree of local adaptation of functionally important Arctic‐alpine biomes by looking at the most diverse taxon of metazoans: the Arthropoda. This is the first study to utilize metabarcoding in the Alpine tundra, providing insights into the effects of micro‐environmental parameters on alpha‐ and beta‐diversity of arthropods in such unique environments. To characterize arthropod diversity, pitfall traps were set at three middle‐alpine sampling sites in the Scandinavian mountain range in Norway during the snow‐free season in 2015. A metabarcoding approach was then used to determine the small‐scale biodiversity patterns of arthropods in the Alpine tundra. All DNA was extracted directly from the preservative EtOH from 27 pitfall traps. In order to identify the controlling environmental conditions, all sampling locations were equipped with automatic data loggers for permanent measurement of the microenvironmental conditions. The variables measured were: air temperature [°C] at 15 cm height, soil temperature [°C] at 15 cm depth, and soil moisture [vol.%] at 15 cm depth. A total of 233 Arthropoda OTUs were identified. The number of unique OTUs found per sampling location (ridge, south‐facing slope, and depression) was generally higher than the OTUs shared between the sampling locations, demonstrating that niche features greatly impact arthropod community structure. Our findings emphasize the fine‐scale heterogeneity of arctic–alpine ecosystems and provide evidence for trait‐based and niche‐driven adaptation. The spatial and temporal differences in arthropod diversity were best explained by soil moisture and soil temperature at the respective locations. Furthermore, our results show that arthropod diversity is underestimated in alpine‐tundra ecosystems using classical approaches and highlight the importance of integrating long‐term functional environmental data and modern taxonomic techniques into biodiversity research to expand our ecological understanding of fine‐ and meso‐scale biogeographical patterns.

## Graphical abstract

Our study examines the alpha‐ and beta‐diversity of arthropods in the Arctic‐alpine biomes of the Scandes using environmental DNA (eDNA)/metabarcoding. We found that micro‐climatological parameters such as air/soil temperature and soil moisture significantly influence the arthropod community structure, highlighting the fine‐scale heterogeneity of these ecosystems. Our study emphasizes the importance of integrating long‐term functional environmental data and modern taxonomic techniques to accurately assess arthropod diversity and broaden our understanding of biogeographical patterns in alpine‐tundra ecosystems.

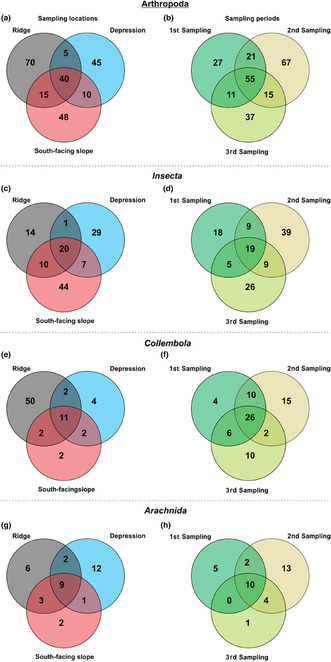

## 1 INTRODUCTION

The Scandinavian mountain range spans across extensive portions of the arctic–alpine ecosystem complex found in the northern hemisphere. Parts of these arctic–alpine ecosystems are often referred to as alpine–tundra. These habitats are unmatched, offering refuge to distinctive species assemblages due to several key factors: (i) their habitat heterogeneity, even over short distances, (ii) a wide range of taxa from persistent pioneer species to successional taxa and ultimately climax taxa (Vater & Matthews, 2013). Despite their critical ecological significance, there remains a considerable knowledge gap regarding the potential impacts of climate change on arctic–alpine ecosystems.

Arctic–alpine ecosystems are one of the regions most affected by climate change (Ernakovich et al., 2014; IPCC, 2022; Macias‐Fauria & Johnson, 2013). The alterations in response to climatic change are already visible at all scales (Alsos et al., 2012; Hobbie et al., 2017; Høye et al., 2014; Kausrud et al., 2008; Parmesan & Yohe, 2003). The impacts of climate change come in addition to the significant loss of insect biomass and insect biodiversity caused by industrialized agriculture (Hallmann et al., 2017; Raven & Wagner, 2021; Wagner, 2020). It has been further suggested that climate change will not only directly impact single species, e.g., through body size changes (Gardner et al., 2011; Høye et al., 2009), but that it will further impact biocoenoses and food webs, e.g., by altering biotic interactions with multiplying effects on ecosystems (Schilthuizen & Kellermann, 2014; Schmidt et al., 2017; Wirta et al., 2015; Zarnetske et al., 2012). The predictions of the concomitant effects of this change in fauna, flora, and resources often lack precision and are still inconsistent. Moreover, studies substantiating these hypotheses are still scarce (Gillespie et al., 2020; Post, 2013; Zarnetske et al., 2012). To better understand ecosystem and organism responses to climate variability, it is imperative to undertake a comprehensive study within their ecological context (Franks & Hoffmann, 2012; Gienapp et al., 2008).

In arctic–alpine ecosystems, steep topographic gradients result in pronounced differences in abiotic factors such as near‐ground air temperature, soil temperature, and soil moisture, which drive local organism ensembles and activity (Frindte et al., 2019; Löffler & Pape, 2020). The changes along these gradients cause heterogeneous fine‐scaled variations in abiotic and biotic conditions across relatively short geographic distances (Nagy & Grabherr, 2009; Scherrer & Körner, 2011; Wundram et al., 2010). This habitat heterogeneity results in relatively high biodiversity levels in mountainous habitats (Badgley et al., 2017; Mosbrugger et al., 2018; Simanonok & Burkle, 2014). This makes arctic–alpine areas good study‐cases for exploring the underlying processes of biodiversity patterns at different scales and how species abundances and diversity change along such environmental gradients (Dehling et al., 2014; Hodkinson, 2005; Sundqvist et al., 2013).

Terrestrial arthropods commonly occur in high abundances, even in relatively cold environments (Blagoev et al., 2013; Danks, 2004; Finch & Löffler, 2010), and are highly sensitive to environmental factors such as season length (Hein et al., 2019; Høye et al., 2009; Legault & Weis, 2013), temperature (Arthofer et al., 2013; Bowden et al., 2015), humidity (Entling et al., 2010), and precipitation (Kaspari et al., 2008). In fact, arthropods are known as biological indicators of environmental variability, and mountain habitats, such as the alpine tundra, are often compared to islands because they provide refuges for species that cannot survive at lower elevations (Graham et al., 2014; Slatyer & Schoville, 2016; Steinbauer et al., 2016). Despite their ecological importance, there are only limited conservation efforts for 75% of the known insects (Chowdhury et al., 2023).

Alpine terrestrial arthropods are valuable proxies in process‐oriented ecological studies (e.g. Bishop et al., 2014; Donnelly et al., 2012; Schilthuizen & Kellermann, 2014). However, ecological studies are often limited to singular taxonomic groups of arthropods, e.g., ants (Kaspari et al., 2008), grasshoppers (Nufio et al., 2010), spiders (Blagoev et al., 2013; Bowden et al., 2015), or mites (Hågvar & Klanderud, 2009). Others are restricted to the analysis of only a single arthropod species, e.g., *Mitopus morio* (Arthofer et al., 2013; Hein et al., 2014) or *Pardosa glacialis* (Høye et al., 2009) Morphological identification is often based on differences in genitalia; hence, most studies rely mainly on imagines. Such approaches can bias biodiversity estimates because the identification of juveniles is challenging—if not impossible—and thus the integration of early life stages in synecological research is fragmented (Violle et al., 2012). Moreover, larval lifestyles may differ dramatically from adult behavior, e.g., regarding diet or preferred habitat (reviewed in Thiele, 2012). Even in well‐studied taxa and regions, morphological identification remains quite time‐consuming and requires taxonomic expertise (cf. Luff & Larsson, 1993), limiting its practicability for ecological studies, which often rely on large datasets. This, coupled with the need to integrate high‐resolution data at multiple scales, has led to rapid automation in field ecology alongside taxonomists' efforts to maintain existing taxonomic data first (Ärje et al., 2020; MacLeod et al., 2010).

There is a pressing need for tools that can expedite and enhance the accuracy of species identification, allowing us to fully harness the potential of arthropod communities as indicators of environmental change and key contributors to ecological processes. Recent developments in metabarcoding studies using high‐throughput sequencing (HTS) have allowed comprehensive biodiversity assessments on whole communities encompassing thousands of species across diverse environments and taxonomic groups, including various life stages (e.g., Fonseca et al., 2010, 2014; Mardis, 2008; Shendure & Ji, 2008). DNA‐based methodologies offer a significantly enhanced capacity for precise taxonomic identification from environmental samples (e.g., Fonseca et al., 2017; Lallias et al., 2015; Zimmermann et al., 2014) and detect orders of magnitude more sequence information compared to traditional Sanger sequencing methods (Haas et al., 2011). While traditional morphology‐based assessments rely on time‐consuming sorting and individual identification of single specimens, a single bulk environmental sample has the potential to yield comprehensive data on an entire community comprised of hundreds of species. HTS costs are strongly decreasing (Shokralla et al., 2012), and the applicability of HTS to large‐scale environmental DNA (eDNA) biodiversity is well‐established, relatively easy, and has increased substantially over the last years (Fonseca et al., 2010, 2023; Hajibabaei et al., 2007; Lallias et al., 2015; Shokralla et al., 2015; Taberlet et al., 2012). Emerging techniques like metabarcoding approaches hold promise in addressing the lingering gaps pertaining to temporal and spatial arthropod diversity within high‐latitude ‘cold‐environments,’ such as arctic–alpine ecosystems (Gillespie et al., 2020; Slatyer et al., 2017). Nonetheless, it is worth noting that these technical advancements may not have been previously evaluated within the distinct biome of the Alpine tundra.

Our primary aim is to assess the suitability of eDNA isolated from fresh ethanol (herein referred to “ethanol‐based DNA”) within pitfall traps as a method for discerning the biodiversity of terrestrial arthropods in bulk samples. The principal objectives encompass the detection of spatial and temporal variations in arctic–alpine arthropod diversity and its environmental drivers to further contribute and enable reliable predictions for conservation and survey planning. This study demonstrates the effectiveness of molecular methods for addressing specific research questions and their capacity to overcome the challenges associated with morphological identification. The application of these methods will facilitate the identification of fine‐scale arthropod biodiversity in the diverse arctic–alpine environments of Central Norway.

## 2 METHODS

### 2.1 Study area

**FIGURE 1:**
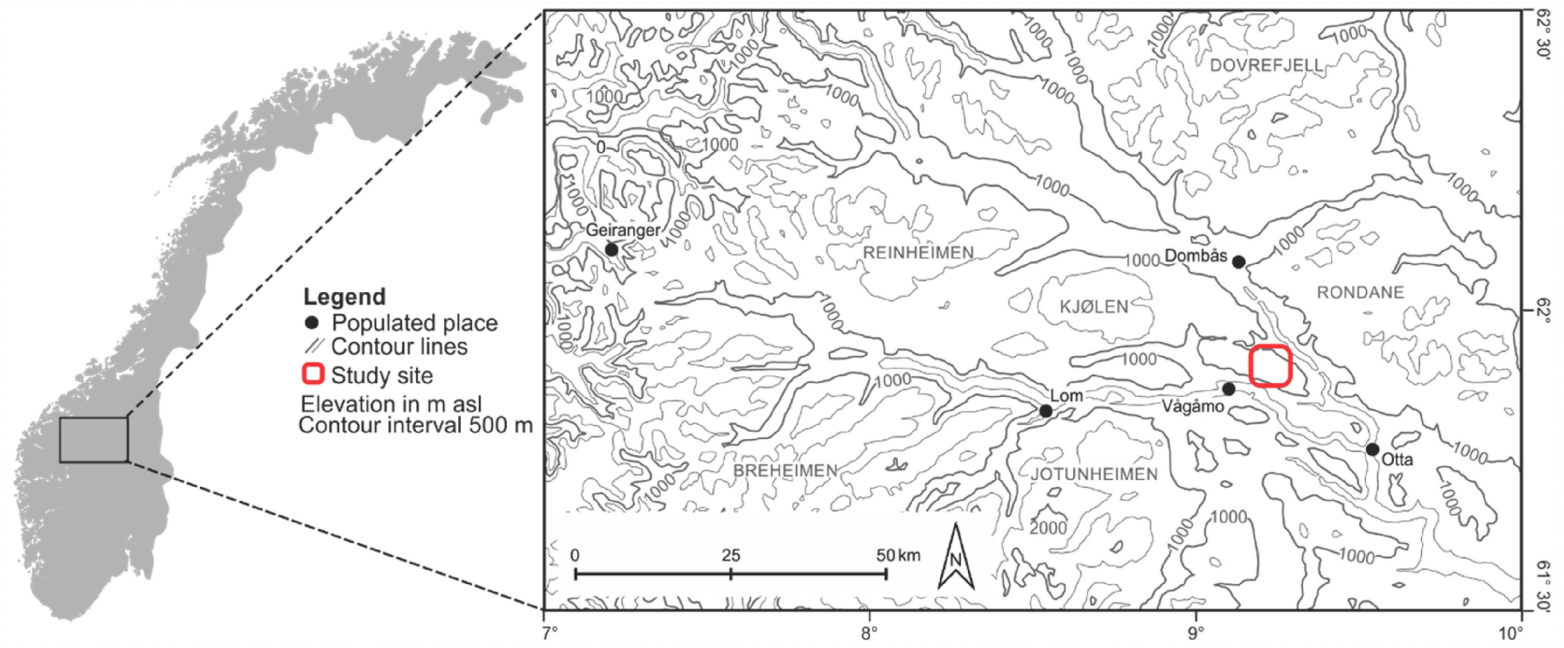
Sampling area in Norway in the most continental part of Central Norway (modified after Hein et al., 2014). This study was conducted at three middle‐alpine sites characterized by pronounced microclimatic differences, a ridge at 1565 m a.s.l. (N 61.9045, E 9.2456 [WGS 84]), a depression at 1480 m a.s.l. (N 61.9005, E 9.2757), and a south‐facing slope at 1534 m a.s.l. (N 61.9040, E 9.2483).

The research area Vågå (Figure 1) is located in the most continental part of Norway (Oppland, 61°53′ N; 9°15′ E). It is characterized by very low annual precipitation values, of 300–600 mm (depending on elevation), and it is one of the most arid places in Norway (Moen, 1998). The annual average temperature at 1000 m above sea level (a.s.l.) is below −2°C, and the average annual temperature in the warmest month is below 10°C. Above the tree line at around 1000 m a.s.l., the low‐alpine belt stretches up to approx. 1400 m a.s.l. From here, the middle‐alpine zone goes upward to the highest peak “Blåhø” at 1618 m a.s.l. The elevational belts are characterized by some typical plant species that show a clear dependence on topography (Löffler & Finch, 2005). *Betula pubescens* forms the tree line of the study area. The low‐alpine belt is characterized by chionophilic (snow‐adapted) shrubs such as *Betula nana*, *Juniperus communis*, *Empetrum nigrum hermaphroditum*, *Vaccinium myrtillus*, *Vaccinium uliginosum*, *Vaccinium vitis‐idaea*, and a variety of *Salix* species. This type of vegetation is typically found in the sheltered locations with large accumulations of snow (primarily south‐exposed slopes). Generally, lichens dominate the vegetation at the exposed ridge sites, e.g. *Alectoria ochroleuca*, *Flavocetraria nivalis*, *Cladina stellaris*, and *Cladina arbuscula*. The middle‐alpine belt is characterized by a more mosaic‐like vegetation pattern. It begins where the European blueberry *Vaccinium myrtillus* disappears from sites with a relatively long snow cover. In general, the middle‐alpine belt is dominated by grassier vegetation, e.g., *Juncus trifidus* and *Luzula confusa*. The distributions of the vegetation types are governed by both microclimatic factors and the topography related snow‐cover duration and thickness (Löffler, 2003; Löffler & Finch, 2005; Pape et al., 2009). This study was conducted at three middle‐alpine sites characterized by pronounced microclimatic differences, a ridge (1565 m a.s.l.), a depression (1480 m a.s.l.), and a south‐facing slope (1534 m a.s.l.).

### 2.2 Micro‐environmental measurements, pitfall trapping, and sampling

In order to characterize arthropod diversity, pitfall traps were set up at the three middle‐alpine sampling sites (*n* = 3 per site), consisting of a ridge, a depression, and a south‐facing slope in Central Norway in Vågå. The three sampling sites were equipped with automatic data loggers (HOBO, 4‐channel micro‐stations) for permanent measurements of the micro‐environmental conditions (Bär et al., 2008; Frindte et al., 2019; Löffler et al., 2006). Data loggers at each sampling location measured continuously (as hourly means) the following micro‐environmental variables: air temperature [°C] at 15 cm height (hereafter referred to as T + 15), soil temperature [°C] at 15 cm depth (hereafter T‐15), and soil moisture [Vol.‐%] at 15 cm depth (hereafter SM‐15). Three pitfall traps were installed in close proximity (~10 m) to the loggers in the ground. The traps were filled with propylene glycol as a preservative. Pitfall traps consisted of glass containers, with a height of 120 mm and with trap openings of 55 mm in diameter, upon which a black plastic ring held a semi‐transparent plastic roof (e.g., Hein et al., 2014; Naujok & Finch, 2004). The pitfall traps were emptied three times on a biweekly basis during summer, starting on 26th July until 6th September of 2015. A total of three sampling periods, each lasting for 14 days, were conducted in the middle‐alpine belt. First sampling period: 26th July–9th August, second sampling period: 9th August–23rd August, and third sampling period: 23rd August–06th September. The sampled material was then transferred and preserved in 96% EtOH and stored at −20°C, until further processing (ca. 6 months). DNA preserved in ethanol encompasses genetic material obtained directly from environmental samples (malaise traps, soil, and water), and it thus could fall under the category of eDNA (Ruppert et al., 2019); nonetheless, for clarity within this context, it will be referred to mainly as “ethanol‐based DNA.”

### 2.3 DNA extraction, metabarcoding amplicon libraries, and high‐throughput sequencing

Total DNA was extracted directly from the preservative EtOH from 27 pitfall traps, following the method proposed by Shokralla et al. (2010). After evaporation of 5 mL preservative ethanol at 56°C, DNA was extracted using the Qiagen Blood and Tissue Kit, following the manufacturer's protocol, adding 5 μL RNAse A during extraction as recommended in the protocol. DNA was eluted in two elution steps, each with 50 μL of AE buffer. All DNA samples were PCR‐amplified using the cytochrome c oxidase subunit I (COI) 313 bp gene region, consisting of the forward primer mlCOIintF (5′‐ACACTCTTTCCCTACACGACGCTCTTCCGATCT GGWACWGGWTGAACWGTWTAYCCYCC‐3′) and the reverse primer dgHCO2198 (5′‐GTGACTGGAGTTCAGACGTGTGCTCTTCCGATCT TAAACTTCAGGGTGACCAAARAAYCA‐3′) (Leray et al., 2013). In bold are the Illumina adapters followed by the primers. All forward and reverse primer combinations were designed to include the Illumina MiSeq 8 nucleotide index‐tags (i5/i7) and adapters (P5/P7). PCR amplification of the COI region was performed with a 2‐step PCR approach. The PCR1 was performed in a total volume of 15 μL per sample, using 7.5 μL of Q5 Hot Start High‐Fidelity 2× Master Mix (NEB), 0.5 μL of each primer (10 μM), 0.5 μL of bovine serum albumin (Thermo Fisher Scientific), 1 μL Sigma H_2_O, and 5 ng/μL of genomic DNA template. The first PCR involved a 2‐min denaturation at 98°C, followed by 20 cycles with 40 s at 98°C, 40 s at 45°C, 30 s at 72°C, and a final extension of 3 min at 72°C. All PCR1 products were purified using the HT ExoSAP‐IT (Thermo Fisher Scientific) following the manufacturer's instructions. To add the Illumina index tag adapters, a second PCR was performed, where the purified PCR1 products were split into two PCR tubes using 7 μL of the purified PCR1 product for each tube. Amplifications were carried out in a total volume of 25 μL with 12.5 μL of Q5 Hot Start High‐Fidelity 2× Master Mix (NEB), 1.2 μL of each index primer (10 μM), 1 μL of bovine serum albumin (Thermo Fisher Scientific), 2 μL Sigma H_2_O, and 7 μL PCR 1 product. The same PCR conditions were used as previously but with an annealing temperature of 55°C. Negative controls (ultrapure water only) were included for all amplification reactions. Subsequently, PCR2 products were visualized on an agarose gel, purified (QIAquick Gel Extraction Kit, Qiagen), and quantified using the Quantus Fluorometer (Promega). All PCR products were diluted to the same concentration (3 ng/μL) and pooled to create one single library (Fonseca & Lallias, 2016). Amplicon‐index generated libraries were sequenced on an Illumina MiSeq platform using the v3 MiSeq kit (300 cycles) following the 2 × 300‐bp paired‐end sequencing protocol.

DNA extracts and specimen vouchers are kept at Leibniz Institute for the Analysis of Biodiversity Change (LIB). DNA extracts are available from the LIB (Museum Koenig, Bonn) (for the respective voucher numbers see Table 1).

**TABLE 1:** DNA extracts and specimen vouchers are kept at LIB Museum Koenig Bonn. DNA extracts are available from the Biobank of the LIB (former Zoological Research Museum A. Koenig [ZFMK]).

ZFMK‐DNA‐FD15075074	ZFMK‐DNA‐FD15075082	ZFMK‐DNA‐FD15075090
ZFMK‐DNA‐FD15075098	ZFMK‐DNA‐FD15075106	ZFMK‐DNA‐FD15075114
ZFMK‐DNA‐FD15075122	ZFMK‐DNA‐FD15075130	ZFMK‐DNA‐FD15075138
ZFMK‐DNA‐FD15075146	ZFMK‐DNA‐FD15075154	ZFMK‐DNA‐FD15075162
ZFMK‐DNA‐FD15075075	ZFMK‐DNA‐FD15075083	ZFMK‐DNA‐FD15075091
ZFMK‐DNA‐FD15075099	ZFMK‐DNA‐FD15075107	ZFMK‐DNA‐FD15075115
ZFMK‐DNA‐FD15075123	ZFMK‐DNA‐FD15075131	ZFMK‐DNA‐FD15075139
ZFMK‐DNA‐FD15075147	ZFMK‐DNA‐FD15075155	ZFMK‐DNA‐FD15075163
ZFMK‐DNA‐FD15075076	ZFMK‐DNA‐FD15075084	ZFMK‐DNA‐FD15075092
ZFMK‐DNA‐FD15075100	ZFMK‐DNA‐FD15075108	ZFMK‐DNA‐FD15075116
ZFMK‐DNA‐FD15075124	ZFMK‐DNA‐FD15075132	ZFMK‐DNA‐FD15075140
ZFMK‐DNA‐FD15075148	ZFMK‐DNA‐FD15075156	ZFMK‐DNA‐FD15075164

### 2.4 Data analysis

Negative controls and blanks were unable to be sequenced due to the absence of enough amplicons for sequencing. Raw reads were analyzed using QIIME 1.5.0 (Caporaso et al., 2010) and trimmed for the presence of Illumina adapter sequences using Cutadapt version 1.2.1 (Martin, 2011). Paired‐end reads were joined for each sample using the default fastq‐join v1.3.1‐1 method (Aronesty, 2013) within the join_paired_ends.py command in QIIME1 v1.5.0 (Caporaso et al., 2010) using default settings. Joined sequences were quality‐filtered using the split_libraries_fastq.py script from QIIME1, setting the “‐‐phred_quality_threshold” to 19 for a stricter filtering approach. Primers were then trimmed from the joined sequences using Cutadapt version 1.2.1 (Martin, 2011), and sequences shorter than 250 bp or longer than 500 bp were discarded. Potential chimeras were identified and discarded using *de novo* (vsearch uchime‐denovo) and reference‐based (vsearch uchime‐ref) approaches with UCHIME (Edgar et al., 2011). For the reference‐based chimera removal approach, the BOLD COI‐5P reference database was used (http://v3.boldsystems.org/). Quality‐filtered, dereplicated, and chimera‐free sequences were clustered into OTUs at a 97% identity threshold with VSEARCH implementing a single‐pass, greedy centroid‐based clustering algorithm. Taxonomic assignment was performed using representative OTUs with MegaBLAST (Zhang et al., 2000) against the BOLD COI‐5P reference database (BOLD_03_2017 reference database of all taxa comprising 3,855,453 entries). Assignments were only considered over a minimum similarity threshold of 98%, covering at least 50% of the query sequence and a maximum *e*‐value of 0.001. OTUs entries not matching these criteria were labeled as ‘not assigned’ (NA).

### 2.5 Ecological analysis

To test for arthropod community differences between the different middle‐alpine biomes, statistical analyses (e.g., Wilcoxon rank tests) were carried out in R (R Core Team, 2018) using the ‘vegan’ package (Oksanen et al., 2019). Arthropod community composition (dis)similarities between sampling locations and periods were analyzed using non‐metric multidimensional scaling (NMDS) based on Bray–Curtis dissimilarities (Legendre & Legendre, 1998), on a presence–absence matrix. The ‘envfit’ function from the vegan package was used to fit and test the significance of the micro‐environmental variables. This function calculates the goodness‐of‐fit values (*R* ^2^) for metadata onto the NMDS ordination plot and their significance using 999 random permutations.

## 3 RESULTS

### 3.1 Biodiversity found in the middle‐alpine belt

**FIGURE 2:**
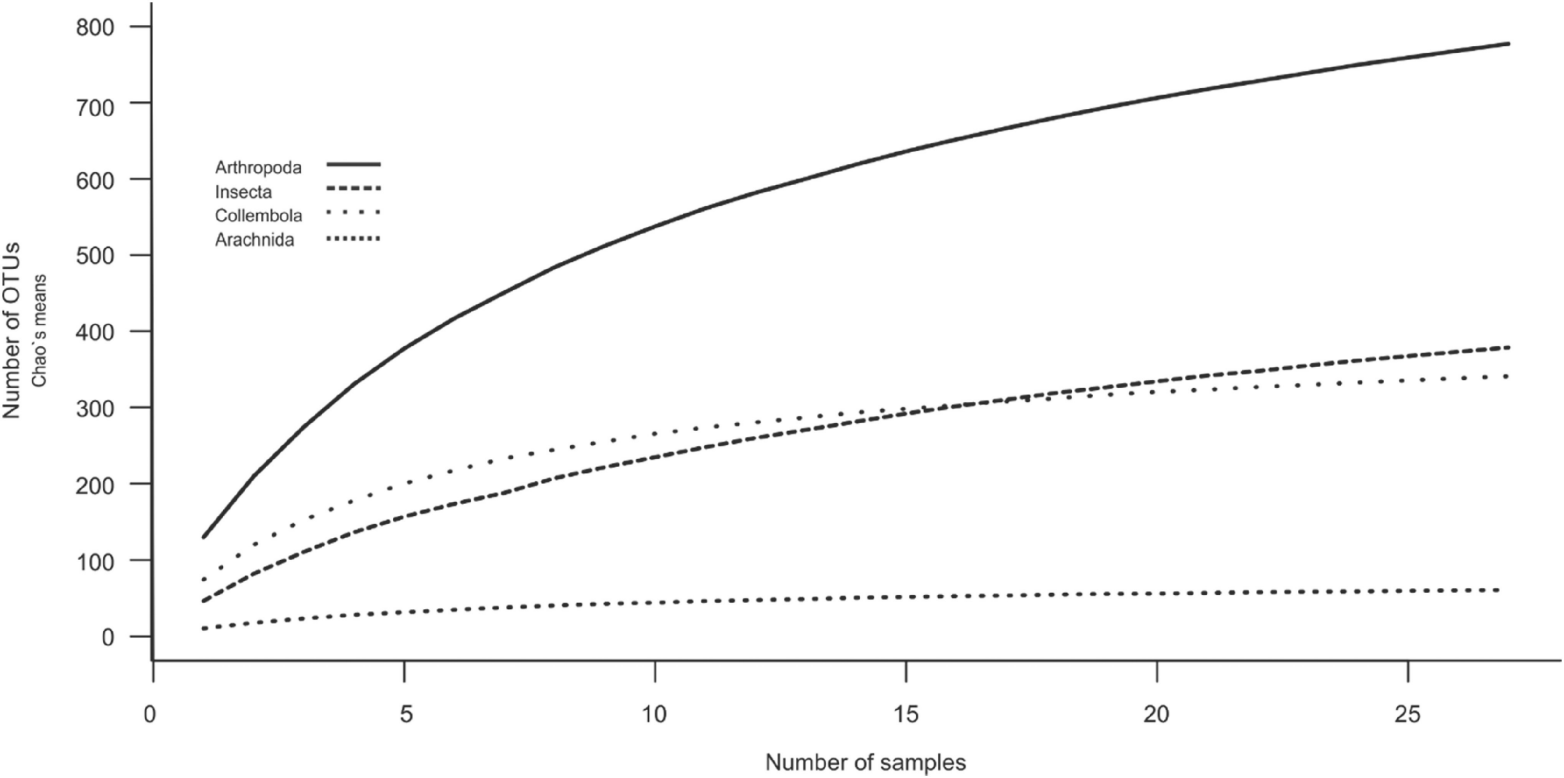
Rarefaction curves for the total number of OTUs (Chao's mean) sampled for the Arthropoda, Insecta, Collembola, and Arachnida. The curves show the cumulated increase in number of OTUs per samples (pitfall traps *n* = 9 and sampling periods *n* = 3, *N* = 27) for each Arthropoda, Insecta, Collembola, and Arachnida.

**FIGURE 3:**
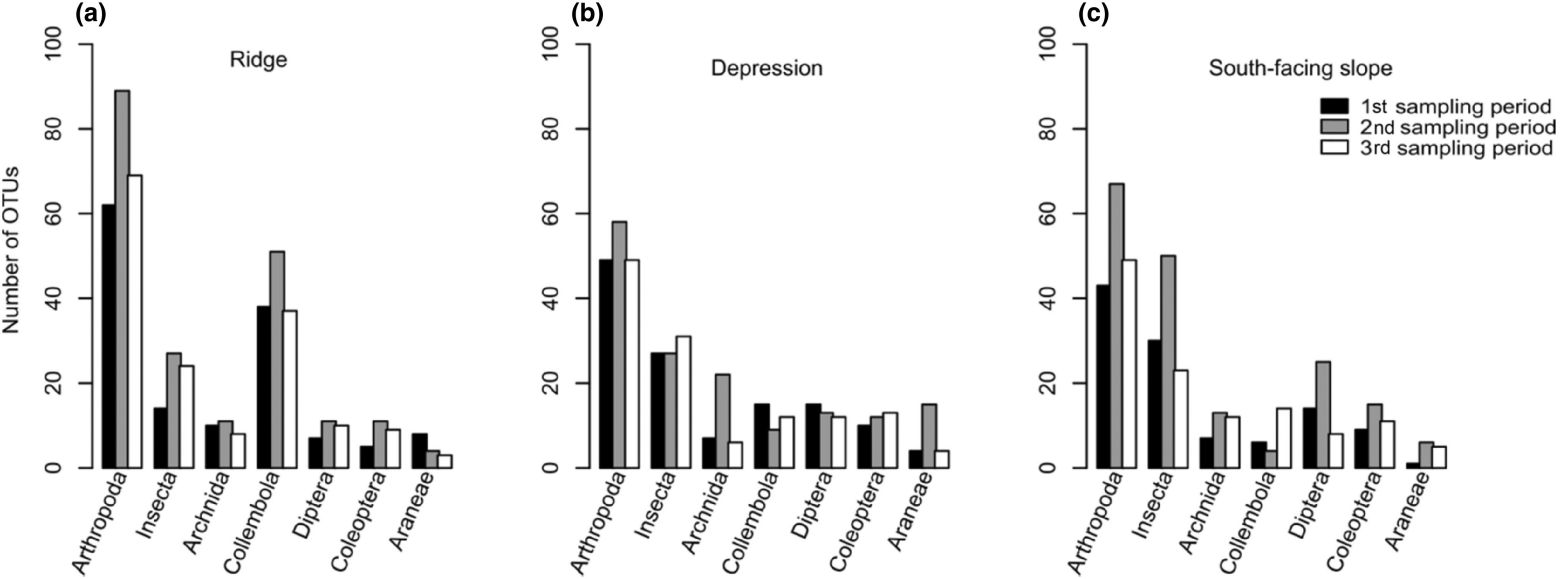
Bar graphs show the number and distribution of Arthropod OTUs at the three sampling sites: (a) ridge, (b) depression, and (c) south‐facing slope and during the three sampling periods (1st sampling period: 26th July–9th August, 2nd sampling period: 9th August–23rd August, 3rd sampling period: 23rd August–06th September). The number of OTUs are shown for Arthropods in general but also for the most common Arthropod classes (Insecta, Arachnida, and Collembola), and orders (Diptera, Coleoptera, and Araneae) in our study.

We identified a total of 5453 OTUs (Operational Taxonomic Units), where 257 OTUs were assigned to Metazoans with a sequence similarity blast hit of at least 98% (Table 2). In total, 233 arthropod OTUs were taxonomically assigned to Arachnida (35 OTUs), Collembola (73 OTUs), and Insecta (125 OTUs)—nine additional OTUs (8× *Locusta migratoria*, 1× *Abax ovalis*) were removed from the analysis due to the likelihood of contamination. Both species, *Abax ovalis* and *Locusta migratoria*, exhibit larger size and were conspicuously absent from our dataset, both in pitfall traps and visual inspections conducted from 2008 to 2016. The inclusion of *Abax ovalis* would constitute the first recorded instance in Norway, and *Locusta migratoria* is non‐native to Norway, commonly used as a food source for terrarium animals. The rarefaction curves indicated an overall good representation of the Arthropods, although still not reaching a plateau (Figure 2). When using 100% sequence similarity BLAST match, a total of 50 OTUs were assigned to Arachnida (four OTUs), Collembola (two OTUs), and Insecta (44 OTUs). For the purpose of this study, OTUs with sequence similarity BLAST matches ≥98% were used as a proxy for species. For a detailed overview of all sampled OTUs with a 98% sequence similarity (BLAST match) at the three sampling locations over the three sampling periods, see Table 2, and for OTUs with a 100% sequence similarity, see Table 3. For Arthropoda, the total number of OTUs ranged between 118–158 and 100–130, during the different sampling periods and location, respectively, whereas for Arachnida, Collembola, and Insecta, the total numbers of OTUs at >98% sequence identity BLAST match varied between 15–76 and 15–81, during the different sampling periods and location, respectively. The sampling period with highest number of OTUs for all taxa was the second sampling period from 9th August to 23rd August (29–158 OTUs), and the sampling location with the highest number of OTUs was the Ridge (20–130) (Table 2). We observed different classes per sampling location (Figure 3), whereby the ridge had the highest number of Arthropod OTUs found over all sampling periods: 62, 89, and 69, compared to 43, 67, and 49 at the south‐facing slope and 49, 58, and 49 at the depression, respectively. Furthermore, a large proportion of the diversity at the ridge is a result of the large numbers of Collembola OTUs 38, 57, and 37, compared to 6, 4, and 14, at the south‐facing slope and 15, 9, and 12 at the depression during the three sampling periods, respectively. Only a small number of OTUs with a BLAST hit of 100% were found during all sampling periods at all topographic locations (*Erigone psychrophila*, *Mycobates tridactylus*, *Entomobrya nivalis*, *Lepidocyrtus lanuginosus*, *Nebria rufescens*, *Patrobus septentrionis*, *Acidota crenata*, *Culex pipiens*, *Spilogona contractifrons*, *Spilogona megastoma*, *Calocoris alpestris*, and *Aporia crataegi*), while various others were unique at one sample location and/or unique at one sample period (Table 2).

**TABLE 2:** Results for the PCR‐based metabarcoding during the three sampling periods (first sampling period: 26th July–9th August, second sampling period: 9th August–23rd August, third sampling period: 23rd August–06th September) at the three different sampling locations (ridge = A, depression = B, south‐facing slope = C).

	Sampling period			Sampling location			All
	1	2	3	A	B	C	
All OTUs	1912	2678	3010	2235	2425	2121	5453
Not assigned (<90% blast hit)	1281	1866	2374	1627	1889	1341	3927
Number of OTUs with at least 90% blast hit							
Total OTUs	628	809	634	605	533	777	1523
Total OTUs including singletons >98%	398	499	456	359	341	497	805
Number of OTUs with at least 98% blast hit							
Arthropoda	114	158	118	130	100	113	233
Arachnida	17	29	15	20	24	15	35
Collembola	46	53	44	65	19	17	73
Insecta	51	76	59	45	57	81	125
Number of OTUs with at least 100% blast hit							
Arthropoda	18	31	30	24	27	30	50
Arachnida	2	4	4	3	3	4	4
Collembola	2	2	2	2	2	2	2
Insecta	14	25	24	19	22	24	44

**TABLE 3:** Results for the PCR based metabarcoding during the three sampling periods (first sampling period: 26th July–9th August, second sampling period: 9th August–23rd August, third sampling period: 23rd August–06th September) at the three different sampling locations (ridge = A, depression = B, south‐facing slope = C). Species names and BIN No. for Arthropods with at least 100% blast hit (x).

Arthropoda	Species	Sampling period			Sampling location		
BIN No.		1	2	3	A	B	C
Arachnida	Araneae						
BOLD:AAD1748	*Erigone psychrophila*	x	x	x	x	x	x
BOLD:AAB5831	*Improphantes complicatus*		x	x	x	x	x
BOLD:AAG9578	*Scotinotylus evansi*		x	x			x
Arachnida	Acari						
BOLD:ACH0172	*Mycobates tridactylus*	x	x	x	x	x	x
Collembola	Entognatha						
BOLD:AAA8924	*Entomobrya nivalis*	x	x	x	x	x	x
BOLD:AAT9557	*Lepidocyrtus lanuginosus*	x	x	x	x	x	x
Insecta	Coleoptera						
BOLD:AAG1804	*Byrrhus fasciatus*		x	x			x
BOLD:ABW8816	*Rhagonycha atra*	x				x	
BOLD:ABW5824	*Nebria rufescens*	x	x	x	x	x	x
BOLD:ABW2229	*Patrobus septentrionis*	x	x	x	x	x	x
BOLD:AAO3110	*Sitona griseus*			x	x		
BOLD:AAS0647	*Acidota crenata*	x	x	x	x	x	x
BOLD:AAN4152	*Anotylus rugosus*			x		x	
BOLD:ABW5328	*Anthophagus alpinus*		x	x		x	x
BOLD:ABX2370	*Mycetoporus boreellus*		x			x	
BOLD:ABW5034	*Olophrum boreale*	x				x	
Insecta	Diptera						
BOLD:AAG2500	*Alliopsis silvestris*			x	x		
BOLD:AAI8808	*Boletina nigricans*		x		x		
BOLD:AAM9243	*Bradysia atroparva*	x		x	x		x
BOLD:ACM6005	*Bradysia normalis*	x					x
BOLD:AAB6579	*Calliphora loewi*		x				x
BOLD:ACJ6363	*Coelosia silvatica*		x		x		
BOLD:ACC5408	*Corynoptera alneti*			x			x
BOLD:ACK9942	*Crumomyia notabilis*		x			x	
BOLD:AAA4751	*Culex pipiens*	x	x	x	x	x	x
BOLD:AAB2384	*Eupeodes curtus*	x			x		
BOLD:ABA3388	*Exechia unimaculata*		x				x
BOLD:AAE3567	*Gymnometriocnemus kamimegavirgus*	x				x	
BOLD:ACJ1377	*Leptosciarella hispida*		x				x
BOLD:AAB9700	*Limnophyes edwardsi*	x				x	
BOLD:AAA6618	*Lucilia sericata*			x	x		
BOLD:ABY5735	*Lycoriella modesta*	x			x		x
BOLD:ABA1215	*Lycoriella sativae*		x	x		x	x
BOLD:ACR4654	*Megaselia diversa*			x		x	x
BOLD:ADA4621	*Megaselia* sp.		x			x	
BOLD:AAE7238	*Micropsectra nana*		x				x
BOLD:AAG7029	*Phaonia subventa*			x	x		
BOLD:ABA3004	*Phronia nigripalpis*		x				x
BOLD:AAH3035	*Pollenia rudis*		x			x	
BOLD:ACE9016	*Scaptomyza pallida*		x	x	x	x	x
BOLD:ACX4405	*Scathophaga furcata*			x	x		
BOLD:ACW5117	*Smittia* sp.			x	x		
BOLD:AAB5278	*Spilogona contractifrons*	x	x	x	x	x	x
BOLD:AAP9046	*Spilogona megastoma*	x	x	x	x	x	x
BOLD:ABW8792	*Tarnania tarnanii*	x	x		x	x	x
Insecta	Hemiptera						
BOLD:AAY9456	*Calocoris alpestris*	x	x	x	x	x	x
BOLD:ABA1021	*Euceraphis punctipennis*	x	x			x	x
BOLD:AAB4894	*Sitobion avenae*		x	x	x		x
Insecta	Hymenoptera						
BOLD:ACF0333	*Pachynematus clibrichellus*			x		x	
Insecta	Lepidoptera						
BOLD:AAA8773	*Aporia crataegi*	x	x	x	x	x	x
BOLD:AAB3454	*Erebia pandrose*		x				x
Insecta	Trichoptera						
BOLD:AAD4327	*Glyphotaelius pellucidus*		x				x
BOLD:AAC6635	*Limnephilus coenosus*			x	x		

During all sampling periods at the ridge, depression, and south‐facing slope locations, the number of OTUs found for the most common Arthropod identified varied between ca. 10 and 90 OTUs (Figure 3). The Insecta dominated within the Depression and South‐facing slope, ranging from 25 to 57 OTUs, whereas the Collembola dominated the Ridge location ranging from 10 to 55 OTUs. The Arachnida, Diptera, and Collembola showed less variable numbers of OTUs throughout all locations, with on average 10–20 OTUs.

**FIGURE 4:**
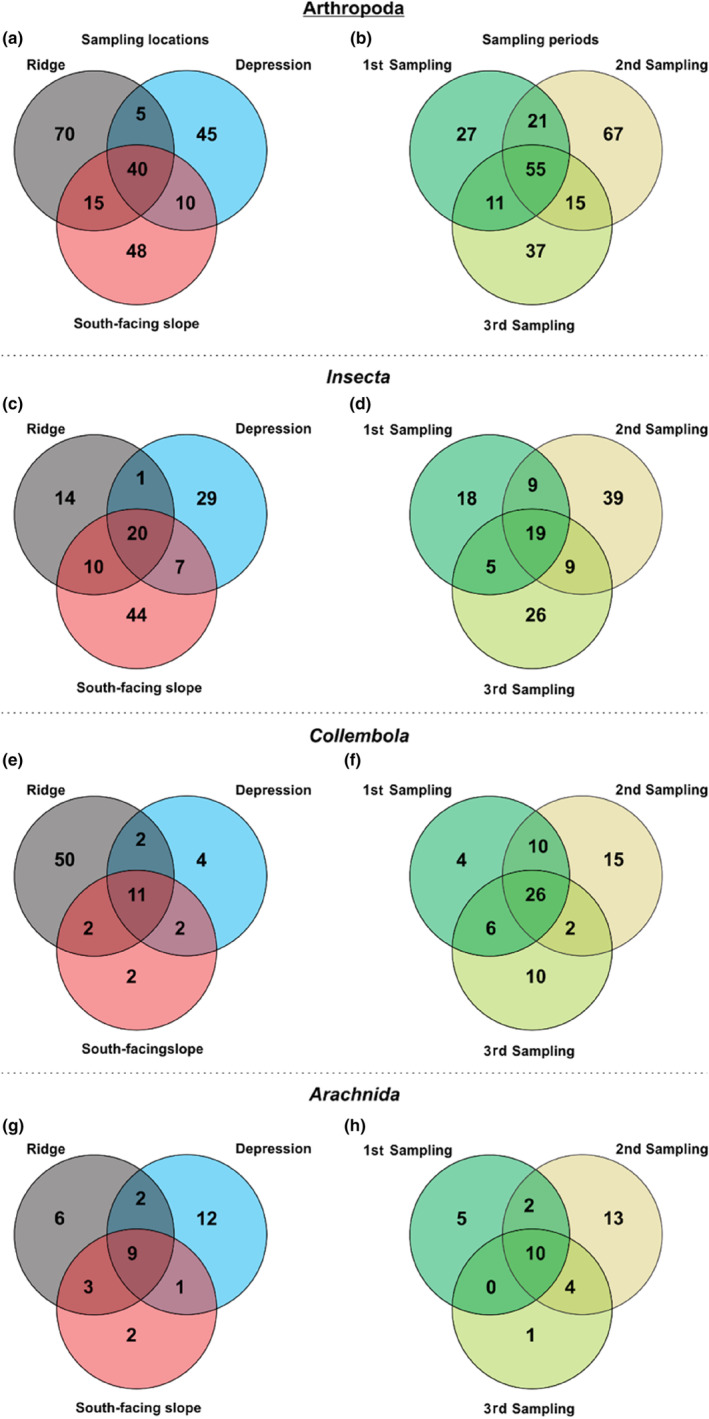
Shared and unique arthropod OTUs (*n* = 233) (with a sequence similarity blast hit ≥98%) sampled in the pitfall traps at the three sampling locations (a) and over three sampling periods in 2015 (b). Additionally, we present the distribution at the three sampling locations and sampling periods for the three main Arthropod groups (c) and (d) Insecta *n* = 125, (e) and (f) Collembola *n* = 73, and (g) and (h) Arachnida *n* = 35, present in the middle‐alpine belt in the continental research area (Vågåmo) in Central Norway. Sampling sites: ridge = gray; depression = blue; south‐facing slope = red. 1st sampling period: 26th July–9th August, 2nd sampling period: 9th August–23rd August, 3rd sampling period: 23rd August–06th September.

Over a period of 6 weeks, the three sampling locations (ridge, depression, and south‐facing slope), showed high numbers of unique arthropod taxa, which only occurred at one of the sampled locations (Figure 4a). The number of shared OTUs between all locations varied from 40, 20, 11, and 9 for the Arthropoda, Insecta, Collembola, and Arachnida, respectively. The ridge showed more unique OTUs for the Arthropoda (70 OTUs) and Collembola (50 OTUs), whereas the depression area showed more unique OTUs for the Insecta (29 OTUs) and Arachnida (12 OTUs). The south‐facing slope showed substantially more unique OTUs for the Arthropoda (48 OTUs) and Insecta (44 OTUs) than for the Collembola and Arachnida (both with two OTUs). When looking at the sampling periods, the second sampling period from 9th to 23rd August always evidenced more unique OTUs for all taxa with 67, 39, 15, and 15 unique OTUs for the Arthropoda, Insecta, Collembola, and Arachnida, respectively. This was followed by the third sampling period from 23rd August to 6th September (37, 26, 10, and 1 OTUs) and the first sampling period from 26th July to 9th August (27, 18, 4, and 5 OTUs) with less unique OTUs for the Arthropoda, Insecta, Collembola, and Arachnida, respectively.

The Arthropods shared 40 OTUs between the three sampling sites, and for all Arthropod classes, the ridge and depression habitats showed the lowest number of shared OTUs (five OTUs). The highest number of unique Arthropod OTUs was observed in the ridge (70 OTUs) and south‐facing slope (48 OTUs), followed by the depression (45 OTUs). The second sampling period had the highest number of unique OTUs (67) for all Arthropod main classes when compared to sampling periods 1 (27) and 3 (37) (Figure 4b,d,f,h).

### 3.2 Micro‐environmental drivers

**FIGURE 5:**
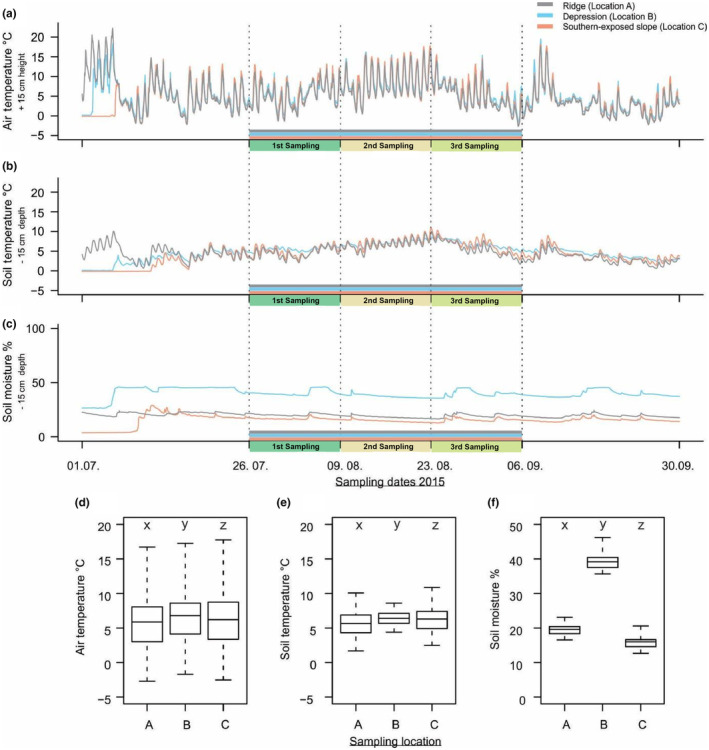
Micro‐environmental conditions during the three sampling periods in the middle‐alpine belt in 2015. Displayed are the air temperatures (°C, +15 cm height, a), soil temperature (°C, −15 cm depth, b), and soil moisture (%, −15 cm depth, c) for the three sampling locations (ridge = gray; depression = blue; south‐facing slope = red) and three sampling periods in focus (1st sampling 9th August; 2nd sampling 23rd August; 3rd sampling 6th September) in the middle‐alpine belt in the continental research area (Vågåmo) in Central Norway. Boxplots showing the differences in air temperature [°C] at +15 cm height (d), soil temperature [°C] at −15 cm depth (e), and soil moisture [%] at −15 cm depth (f) measured at the three sampling locations (A = ridge, B = depression, C = south‐facing slope) during the sampling periods 26th July–6th September. The letters *x*, *y*, and *z* indicate significant differences between the environmental variables at the sampling locations based on two‐sided Wilcoxon rank sum tests (*p* < .05).

Measurement of micro‐environmental conditions at the specific sampling sites showed differences in air and soil temperatures. Nonetheless, more pronounced differences were observed in soil moisture between the three sampling locations and periods (Table 4 and Figure 5a–c). The environmental conditions observed were significantly different between the sites (Wilcoxon rank‐sum, *p* < .05) (Figure 5d–f).

**TABLE 4:** Micro‐climatic data for the three sampling locations (ridge = A, depression = B, south‐facing slope = C) between 26th July and 06th September. Given is the min, max, and mean for air temperature (°C), soil temperature (°C), and soil moisture (%).

Location	Micro‐climatic data								
	Air temperature [°C]			Soil temperature [°C]			Soil moisture [%]		
	Min	Max	Mean	Min	Max	Mean	Min	Max	Mean
Ridge = A	−2.7	16.7	5.9	1.7	10.1	5.6	17	23	19
Depression = B	−1.7	17.3	6.8	4.4	8.6	6.4	36	46	39
South‐facing slope = C	−2.5	17.8	6.4	2.5	10.1	6.2	13	21	16

**FIGURE 6:**
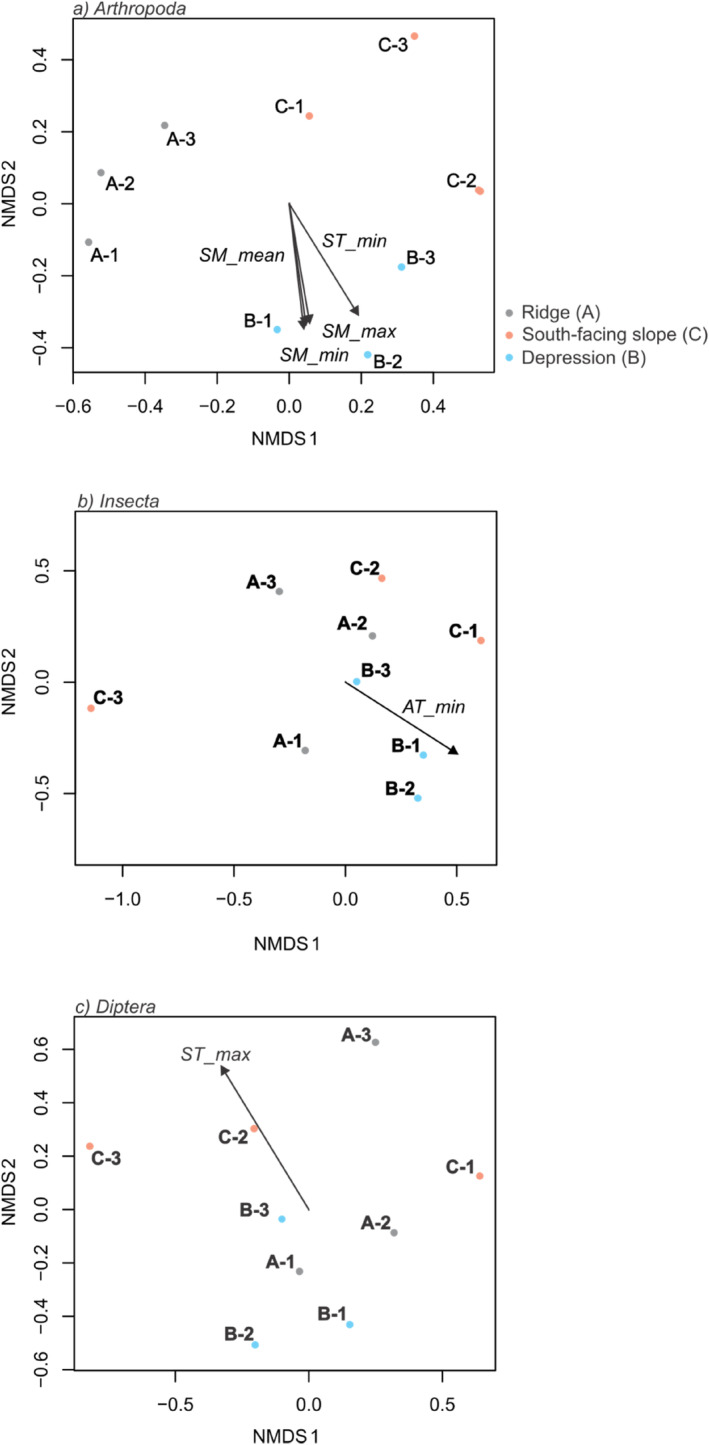
Non‐metric multidimensional scaling (NMDS) plot of (a) Arthropoda, (b) Insecta, and (c) Diptera diversity found at Vågå, Norway. Points represent the three sampling locations (A = ridge, B = depression, and C = south‐facing slope) and three sampling periods (1–3). The arrows indicate significant correlations with different micro‐environmental variables (AT = air temperature, SM = soil moisture, and ST = soil temperature) using the envfit function, displayed are only significant correlations *p* < .05 (Arthropoda: *SM_mean R* ^2^ = .73, *SM_min R* ^2^ = .77, *SM_max R* ^2^ = .71, *ST_min R* ^2^ = .83; (b) Insecta: *AT_min R* ^2^ = .71; (c) Diptera: *ST_max R* ^2^ = .70).

Non‐metric multidimensional scaling (nMDS) performed to identify drivers of arthropod community composition indicated a strong influence of soil temperature (ST) and soil moisture (SM) content on arthropod diversity (Figure 6a). This analysis further showed that each sampling site clustered together independently of the sampling period and soil moisture and was significantly correlated with arthropod community structure (envfit for SM‐mean *R* ^2^ = .73, SM‐min *R* ^2^ = .77, SM‐max *R* ^2^ = .71, ST‐min *R* ^2^ = .83, *p*‐value <.05 respectively) (Figure 6a). Additionally, an nMDS analysis was performed for the three main Arthropod classes (Arachnida, Collembola, and Insecta) and four main orders (Araneae, Coleoptera, Entomobryomorpha, and Diptera), which resulted in significant correlations between the Insecta and air temperature (AT‐min *R* ^2^ = .71, *p*‐value <.05, Figure 6b) and the Diptera and maximum soil temperature (ST‐max *R* ^2^ = .70, *p*‐value <.05, Figure 6c). The other taxa groups showed no significant correlation between the sampling period and location, with community structure and the micro‐environmental variables measured (stat test, *p* > .05).

## 4 DISCUSSION

This study demonstrates the capacity of metabarcoding tools to distinguish arthropod communities, even when they are in close spatial proximity. Furthermore, it underscores the capability of ethanol‐based DNA and other DNA‐based methods to elucidate crucial ecological life‐history traits and fitness factors, which can shed light on cryptic parts of diversity and allow for distinctions in larval developmental times through monitoring.

The use of molecular tools, such as metabarcoding revealed higher than expected biodiversity values for such unique biomes in the Alpine tundra. For example, Pentinsaari et al. (2020) identified 1264 arthropod BINs (unique Barcode Identifiers) with pitfall traps in the Arctic Tundra using barcoding. Additionally, Nørgaard et al. (2021) found that eDNA metabarcoding detected an additional 25 orders and 42 species compared to field observations. This aligns with the outcomes of our metabarcoding study, where we successfully identified 233 arthropod species from the preservative fresh ethanol collected from nine pitfall traps across three sampling periods (*N* = 27). This observation strongly implies that conventional approaches would likely miss a significant portion of the arthropod diversity in this region. However, it is important to point out that our results also illustrate the incompleteness of our understanding of arthropod diversity in this arctic–alpine region, as shown by the rarefaction curves, which do not reach the plateau apart from the Arachnida. While it is important to acknowledge that various factors can contribute to the underestimation of diversity levels in environmental samples (Fonseca, 2018; Kirse et al., 2021b), it is worth considering that the presence of preservative solutions in numerous arthropod traps may potentially hinder the success of downstream PCR amplification or DNA extractions, stemming from less‐than‐optimal preservation conditions (Zenker et al., 2020). In our study, we adopted the practice of discarding the preservative solution (propylene glycol) and instead placed the specimens in fresh ethanol for approximately 6 months. Factors like preservation time and scleroticization can influence the success of PCR amplification in larger individuals (Marquina et al., 2019). The comprehensive assessment of diversity levels in metabarcoding studies can significantly benefit from adopting a multigene approach (Fonseca et al., 2022, 2023; Martins et al., 2021). This approach can substantially augment taxonomic resolution while also broadening the coverage of detected species (Fonseca et al., 2022). In situations where budget constraints are not a limiting factor, integrating multiple methods can further bolster the robustness of these assessments. This is especially relevant in the context of arthropod studies, where leveraging a combination of techniques proves particularly advantageous (Marquina et al., 2019; Martins et al., 2021). One such complementary strategy involves the concurrent utilization of homogenate samples alongside preservative ethanol samples. This tandem approach harnesses the strengths of both methods, allowing for a more comprehensive understanding of the arthropod diversity within an ecosystem (Martins et al., 2021). While preservative ethanol samples are valuable for capturing eDNA or ethanol‐based DNA, a comprehensive understanding of the community is achieved through a combination of metabarcoding methods, including whole organism approaches (Marquina et al., 2019). Amalgamating these approaches leads to a more accurate and nuanced characterization of arthropod diversity within their study area. Furthermore, other factors contributing to incomplete diversity representation might be associated with the notably high alpha‐diversity levels within this region, potentially necessitating a greater number of biological replicates (Bruce et al., 2021; Fonseca et al., 2023). The underrepresentation of taxa from less studied and remote areas (Fonseca et al., 2022), like arctic–alpine ecosystems, within existing databases, coupled with a low sampling effort, might further contribute significantly to the underestimation of diversity. Our findings strongly evidence that metabarcoding analyses of pitfall trap samples provide a cost‐effective and expedited means for detecting terrestrial arthropod biodiversity, as previously demonstrated by studies like Dopheide et al. (2019). However, one of the biggest limitations remains the lack of absolute abundance data, which can lead to a biased evenness of the targeted taxa, particularly at the species‐level. For instance, in the study conducted by Beckers et al. (2018), the Carabid beetles *Nebria rufescens* and *Patrobus septentrionis* exhibited their highest activity and abundances in depression and slope habitats. However, it is important to note that our present study does not allow us to draw definitive conclusions regarding such niche segregation based on our results.

Our results evidence that substantial portions of the sampled arthropod populations exhibit a high degree of endemism and are strongly influenced by niche‐specific environmental factors within arctic–alpine ecosystems. This assertion is supported by the consistently greater number of unique OTUs observed compared to the number of shared OTUs across all habitats. Even though pitfall traps are known to be biased toward more active/vagile species or specimens (commonly males during periods of mating), they provide valuable and reliable means in arctic–alpine ecosystems when sampling ground‐dwelling arthropods (Finch & Löffler, 2010; Hein et al., 2014). Arthropod diversity in our sampling area evidenced highly variable diversity levels, both spatially and temporally, during the arctic–alpine summer season. The emerging patterns of arthropod diversity and relative abundances were significantly correlated to the micro‐environmental conditions. Especially soil moisture and soil temperature had a significant influence on arthropod diversity. It is well‐established that most arthropods (also species with flying imagines) spend at least one stage of their life cycle on or within the soil (Kirse et al., 2021a), and concomitantly soil temperatures have a huge influence on the speed and timing of development of arthropods (Howe, 1967). Soil moisture is one of the key factors, besides temperature and photoperiod, determining arthropod seasonal ecology (Ottesen, 1996; Tauber et al., 1998), even though the direct effect of soil moisture on arthropods is sometimes difficult to decipher (Coulson et al., 2003; Hodek, 2003). The analyses which aimed at identifying micro‐environmental variables as drivers of insect diversity also revealed that minimum air temperature was the most important explanatory factor for the observed variation. Conversely, dipteran diversity exhibited a stronger correlation with soil temperature. This correlation was substantiated through visual inspections conducted prior to wet lab processing, which, in conjunction with findings from prior studies, confirmed the substantial presence of dipteran imagines in the traps. Furthermore, we observed a minor but discernible quantity of dipteran larvae. It is also plausible that dipteran material was introduced into the traps as gut contents, possibly via predation by other organisms, such as spiders. It is worth noting that in various arthropod taxa, soil moisture has been identified as a significant controlling physical factor, e.g., development and mortality rate (Lapointe & Shapiro, 1999; Zheng & Li, 2013), supercooling capacity (Hou et al., 2009), and activity and abundance (Hayward et al., 2004). Our findings align with this notion, as the arthropod assemblages displayed noticeable variations over relatively short distances along the environmental gradient, from ridge to depression habitats. This is evidenced by the large number of unique Arthropod OTUs found at the sampling sites, as a result of the significant differences in fine‐scale environmental conditions. Soil moisture in the arctic–alpine ecosystems is the result of complex interactions regarding climate, microclimate, and abiotic conditions, e.g., topography, soil type, and irradiation angle (Körner, 2007; Nagy & Grabherr, 2009; Pape et al., 2009). The topographic position of a certain sampling site controls snow‐cover duration and thickness and thus year‐round soil‐moisture values (Löffler, 2005; Löffler & Finch, 2005). Along the topographical gradient from ridge to depression, winter conditions are characterized by thick snow cover in the depressions and south‐facing slopes and more or less snow‐free ridges. These snow cover patterns result in pronounced differences in season length and in characteristic vegetation patterns (from dry to wet), such as lichen–heath communities at ridges, shrub and heather communities at slope sites, and bryophytes in the depressions. Previous studies conducted in the same area have indicated that fine‐scale heterogeneity is likewise reflected in spider diversity (Hein et al., 2014, 2019) and to some extent carabid beetle diversity (Beckers et al., 2018). Our findings align with these particular patterns within the phylum Arthropoda. On one hand, only a limited number of highly mobile and generalist species were present in all sampling locations and during all sampling periods: e.g., the cosmopolitan springtail *Entomobrya nivalis*, the mosquito *Culex pipiens*, the Staphylinid *Acidota crenata*, or the butterfly *Aporia crataegi*. On the other hand, one can find various examples for species that are stenotopic, such as typical for wet/humid conditions the two Coleoptera species *Anotylus rugosus* and *Rhagonycha atra*, and *Pachynematus clibrichellus* (Hymenoptera), whose larvae feed on *Carex* sp. However, we assume that in the Alpine tundra, generalist species will benefit from climate warming, while more stenotopic and locally abundant specialist species might struggle in such climates (Hein et al., 2018). This is in line with other findings from the research area and other ‘cold’ environments (Ameline et al., 2018; Beckers et al., 2020; Måsviken et al., 2023). Accordingly, an increase in the variability of the micro‐climatic conditions (e.g., droughts or frost events) as predicted in various climate change scenarios will lead to alterations in the occurrence and distribution of arthropods at different spatial and temporal scales in arctic–alpine ecosystems in Scandinavia.

By combining metabarcoding with long‐term biogeographic approaches, we can enhance our ability to identify the pivotal micro‐environmental factors influencing arthropod diversity patterns across different scales. This integrated approach aids in the assessment of resilience traits within arctic–alpine ecosystems in response to climatic variations. Furthermore, it is essential for such studies to encompass an examination of fitness and competition dynamics within these ecosystems to gain a deeper understanding of how biodiversity patterns evolve. Our study establishes that metabarcoding effectively detects arthropod diversity patterns within bulk samples obtained from alpine–tundra environments. These findings lay the groundwork for long‐term investigations into the transformations occurring within arctic–alpine ecosystems in response to climate change. The adoption of metabarcoding techniques mitigates several challenges associated with the morphological identification of numerous arthropod species. This is particularly useful in species conservation and/or spatial planning processes where traditional taxonomic work is too time‐consuming and/or requires hard‐to‐find expertise, often resulting in invertebrates being underrepresented in planning and conservation efforts. However, research also indicates that by integrating both morphology and metbarcoding tools, taxonomic resolution can be enhanced by up to 30% in monitoring surveys (Pereira et al., 2021).

As a consequential step in characterizing diversity and generating species lists from large‐scale environmental studies, the establishment of ecological networks becomes imperative. These networks aim to forge connections between ecosystem structure, potential functioning, and taxonomic diversity. The cornerstone of such networks should be standardized methods and protocols designed for consistent, multi‐decade operation. They should be built upon recurrent landscape elements, such as ridges, depressions, and north‐/south‐facing slopes. The inclusion of standardized trapping methods, (e.g., Global Malaise Trap Program) and the integration of diverse techniques (e.g., COBRA protocol, Cardoso, 2009) are fundamental in reliably assessing the biodiversity of a given system. These comprehensive protocols should encompass field sampling, capturing both biotic and abiotic factors, meticulous laboratory processing of samples, and robust sample storage practices, which may include voucher collections and biobanking. This approach paves the way for an in‐depth analysis of changes in arthropod diversity within the Alpine tundra—an analysis inextricably linked to the influence of climate change. Furthermore, these networks can serve as guiding lights, offering instructions for future action and conservation efforts. It is our expectation that the current estimation of arthropod diversity in these seemingly ‘cold’ yet ecologically heterogeneous environments vastly underestimates the true richness of species. Moreover, the climatically driven changes are likely to usher in a ‘homogenization’ of the organismal inventory, favoring generalist species over specialized ones at fine and intermediate scales.

## AUTHOR CONTRIBUTIONS

**Nils Hein:** Conceptualization (equal); data curation (equal); formal analysis (equal); investigation (equal); methodology (equal); validation (equal); visualization (equal); writing – original draft (equal). **Jonas J. Astrin:** Conceptualization (equal); methodology (equal); resources (equal); validation (equal); writing – review and editing (equal). **Niklas Beckers:** Investigation (equal); methodology (equal); validation (equal); writing – review and editing (equal). **Hendrik Giebner:** Data curation (equal); formal analysis (equal); investigation (equal); software (equal); visualization (equal); writing – review and editing (equal). **Kathrin Langen:** Formal analysis (equal); validation (equal); writing – review and editing (equal). **Jörg Löffler:** Conceptualization (equal); data curation (equal); formal analysis (equal); investigation (equal); methodology (equal); project administration (equal); resources (equal); validation (equal); visualization (equal); writing – review and editing (equal). **Bernhard Misof:** Conceptualization (equal); methodology (equal); project administration (equal); resources (equal); writing – review and editing (equal). **Vera G. Fonseca:** Conceptualization (equal); data curation (equal); formal analysis (equal); methodology (equal); resources (equal); software (equal); validation (equal); writing – original draft (equal).

## FUNDING INFORMATION

This research was partially funded by the German Federal Ministry of Education and Research, through the project German Barcode of Life (Grant number 01LI1101 and 01LI1501).

## CONFLICT OF INTEREST STATEMENT

The authors declare no conflict of interest.

## Supporting information


Appendix S1.

## Data Availability

The climatic data underlying this article are available in the Dryad Digital Repository at https://doi.org/10.5061/dryad.qjq2bvqmh. The sequence data underlying this article are available the Sequence Read Archive (SRA) (Bioproject PRJNA956470, Submission SUB13087141) https://www.ncbi.nlm.nih.gov/sra. The data are not yet published but can be downloaded from the following link: https://datadryad.org/stash/share/G8cM1N6NyuWVAVwvXPEYjwU_4cP8rJ7Z_L3oQG88ook.
